# Increased Strip Width Balancing Interspecific Competition and Enhanced Yield in Maize–Flax Intercropping Systems

**DOI:** 10.3390/plants15192895

**Published:** 2026-09-22

**Authors:** Yifan Wang, Jing Han, Xinkang Ma, Bing Wu, Bin Yan, Peina Lu, Yuhong Gao

**Affiliations:** 1 Gansu Provincial Key Laboratory of Aridland Crop Science, Lanzhou 730070, China; 2 College of Agronomy, Gansu Agricultural University, Lanzhou 730070, China; 3 College of Life Science and Technology, Gansu Agricultural University, Lanzhou 730070, China

**Keywords:** grain yield, yields components, dry matter, relative crowding coefficient, recovery effect

## Abstract

Interspecies interaction is key to achieving intercropping advantages. However, in semi-arid rainfed agricultural areas, research on how strip width influences interspecific relationships and system productivity in maize–flax intercropping remains insufficient. A three-year field experiment was conducted to assess the impacts of varying strip widths (80, 120, and 160 cm) on system productivity and interspecies interactions in a maize–flax intercropping system. The results demonstrated that the 160 cm strip-width treatment achieved the highest land equivalent ratio (LER) and total system yield, with average increases of 11.3% and 37.5%, 7.3% and 38.8% compared to the 120 cm and 80 cm strip-width treatments, respectively. The improvement in grain yield was primarily attributed to synergistic increases in maize kernel number per ear and 1000-kernel weight, as well as enhanced capsule number per plant, seeds per capsule, and 1000-seed weight of flax. Analysis of interspecific competition dynamics revealed that flax exhibited stronger competitive ability during its later growth stages (kernel-filling and maturity stage) under the 160 cm strip width, with an average increase of 97.2% compared to other treatments. Furthermore, the wider strip width optimized the relative crowding coefficient (RCC) and promoted the recovery effect of maize (RE) after flax harvest. Correlation analysis indicated a significant negative relationship between the intensity of interspecific competition during the maize V3 and V12 stages and flax yield. In conclusion, adopting a 160 cm strip width in the maize–flax intercropping system maximizes overall productivity by optimizing yield components, promoting dry matter accumulation, and balancing interspecific interactions.

## 1. Introduction

Intercropping, which refers to the agricultural practice of cultivating two or more crop species concurrently on the same plot of land, constitutes an essential cropping strategy, especially for small-scale and organic farming systems across various regions globally [1,2]. In recent years, intercropping has garnered increasing scholarly and practical attention, primarily owing to its demonstrable agroecological advantages over conventional monoculture systems [3,4]. The most notable benefit of intercropping is the potential to achieve a greater yield or income through the more efficient utilization of agricultural resources, including nutrients, water, heat, and radiation [5]. Furthermore, the practice of intercropping has been demonstrated to mitigate soil erosion, diminish the prevalence of plant diseases, insects, and weeds. Accordingly, it has been perceived as a potential alternative for sustainable agricultural practices.

Interspecific interactions are an unavoidable consequence in strip intercropping systems, in which two crops are cultivated in proximity to one another. The two principal types of interspecific interactions are competition and facilitation. In the intercropping system, both interspecific competition and facilitation between the various crop species coexist. If the facilitation is greater than the competition, the intercropping system will demonstrate an advantage, and vice versa [6]. Early research indicated that competition is the most significant interaction between component intercrops [7]. Furthermore, recent studies have shown that the high yield of intercropping stems from compensatory growth of late-maturing crops, despite early-maturing crops negatively affecting less competitive species (e.g., late-maturing intercrops) during co-growth periods [8,9]. It is therefore very important to regulate the interspecific relationship between early- and late-maturing crops by optimizing agronomic management measures.

The phenomenon of interspecific competition has been observed to promote the utilization of different resources, with intercrops employing a given resource based on ecological niche differences inherent to species involved [10]. Strip configuration is one of the important bases for the occurrence of intercropping interaction [11]. Optimal strip configuration enhances light energy distribution across the crop canopy [12]. Moreover, intercropping involves the fine-scale spatial mixing of different species, which facilitates strong interspecific interactions and maximizes the benefits derived from adjacent heterospecific plant interactions, such as enhanced nutrient uptake [13,14]. However, these positive effects are not universal across time or space. In practice, intercropping can also impose competitive disadvantages on weaker species, which may be outcompeted by taller or fast-growing neighbors for light and nutrients, leading to reduced growth or yield [15]. Such asymmetric competition may undermine overall productivity, particularly under resource-limited conditions [16]. Thus, the success of intercropping is context-dependent, and careful species selection, spatial design, and temporal scheduling are essential to minimize negative interactions and realize intended benefits.

Intercropping boosts land-use efficiency, diversifies production for income stabilization, and reduces input costs by decreasing synthetic fertilizer and pesticide applications [17]. Economically, however, an intercropping system presents notable disadvantages: greater labor inputs for planting and harvesting, and possible yield penalties for subordinate crops when interspecific competition is poorly managed [18]. Therefore, rational regulation of crop spatial configuration and strip width to adapt to mechanized cultivation is a critical prerequisite for reducing production costs and improving the practical applicability of intercropping systems. In an intercropping system involving maize, taller maize will preferentially capture sunlight due to its higher ecological niche, which contributes to increased biomass and grain yield [19]. Conversely, the interception and utilization of sunlight by lower crops will be reduced due to the shading effect [20]. In maize/soybean intercropping system, the optimum strip width was 200 cm for obtaining high grain yields, which improved the competitive ability of soybean by maintaining the competitive ability of maize [21]. Another study showed that the effect of soybean strip width on leaf PAR interception was greater than that of maize strip width in maize and soybean intercropping system [22]. There is a shortage of mechanism studies on effects of intercropping system yield benefits of increased strip width.

Maize–flax intercropping represents a widely adopted practice in the Loess Plateau and other semi-arid regions of Northwest China, where maize (*Zea mays* L.) and flax (*Linum usitatissimum* L.) are cultivated as major cash and oilseed crops, respectively [23]. In this intercropping system, tall maize canopy provides shading that reduces soil evaporation, while the combination of deep-rooted maize and fibrous-rooted flax allows complementary water uptake from different soil layers, thereby enhancing overall water use efficiency in these water-limited environments [24]. However, traditional maize–flax intercropping systems with narrow strip configurations (e.g., 80 cm:80 cm) intensify interspecific competition [24], which not only suppresses the growth of the flax but also impedes mechanized field operations. Although widening strip width provides a viable option for mechanized field management, it expands row spacing between maize and flax. The resultant decline in interspecific interactions within the intercropping system may exert adverse effects on total system yield performance [11]. Therefore, optimizing strip width is essential to balance interspecific interactions with mechanical operability; however, quantitative studies addressing this trade-off in maize–flax intercropping remain scarce, particularly in the dryland regions of Northwest China.

Therefore, the objective of the present study was to evaluate the effects of maize/flax intercropping on different strip width on maize and flax growth, yield performance and interspecific interaction effects in intercropping systems. We hypothesize that (a) reasonable strip-width configuration enhances the yield performance of the maize/flax intercropping system; (b) appropriately widened strip width can promote dry matter accumulation, optimize yield components, and further improve the land equivalent ratio (LER) of the intercropping system; (c) widening the strip width will alleviate interspecific competition between maize and flax by increasing their physical distance, thereby improving flax growth and yield.

## 2. Results

### 2.1. Grain Yield, Biological Yield, Land Equivalent Ratios and HI

Intercropping significantly increased the grain yield of crops compared to monoculture (Table 1). Under the same land area, the grain yield of maize in M4F8 treatment increased by an average of 5.7% relative to monoculture. The total system yield of the intercropping system was 4.5% higher than the weighted average of monoculture maize and flax.

In the intercropping system, both grain yield and biomass yield increased with the increase in the width of the strips (Table 1). Under the 160 cm strip-width configuration (M4F8), the grain yield of the intercropping system reached the highest, increasing the maize, flax and total system yield by 6.4% and 38.6%, 19.6% and 40.7%, and 7.3% and 38.8% respectively compared to the 120 cm (M3F6) and 80 cm (M2F4) strip-width treatments. The 120 cm strip width significantly increased the grain yield and total system yield of maize and flax by 30.3%, 17.7% and 29.2% respectively compared to the 80 cm strip-width treatment. The biomass yield showed that under the 160 cm strip width, the maize, flax and total biomass yield increased by 15.3%, 21.2% and 16.0% respectively compared to the 80 cm strip-width treatment, and there was no significant difference compared to the 120 cm strip-width treatment.

All intercropping systems have a land equivalent ratio (LER) lager than 1, indicating intercropping outperforms sole cropping systems (Figure 1). Over a three-year period, the 160 cm strip-width configuration treatment led to an 11.3% higher LER in intercropping systems compared to the 120 cm strip-width configuration treatment, and a 37.5% improvement relative to the 80 cm strip-width configuration treatment. The 120 cm strip-width configuration also enhanced the LER by 23.5% when compared to the narrowest 80 cm strip-width configuration treatment. The partial land equivalent ratio (PLER) of maize was the highest under the 160 cm bandwidth treatment and showed no significant difference from the 120 cm bandwidth treatment, with an average of 1.07 over three years, which was 36.9% higher than the 80 cm bandwidth treatment. There was no significant difference in the land equivalent ratio of flax among the treatments.

The HI of maize exhibited significant interannual variability, with no discernible distinction observed between intercropping and monoculture treatments (Figure 2). The HI of flax was higher in intercropping systems than in monoculture, except in 2022, showing an average increase of 20.1% under intercropping compared to monoculture. Within the intercropping treatments, the 160 cm bandwidth treatment significantly increased the HI of flax by 10.0% and 17.6% compared to the 120 cm and 80 cm bandwidth treatments, respectively. Overall, the HI of maize was significantly higher than that of flax in the intercropping system, with an average increase of 67.3% relative to flax.

### 2.2. Yields Components

Over the three-year period, the average number of maize ears under intercropping showed a significant increase, rising by 27.8% compared to monoculture (Table 2). Intercropped flax treatment significantly enhanced capsule number, seed number per capsule, and 1000-seed weight by 51.4%, 20.6%, and 13.6%, respectively, compared to monoculture (Table 3).

In the intercropping system, the ear number of maize was the highest in the 80 cm strip-width treatment, which was 19.0% higher than that in the 120 cm bandwidth, and there was no significant difference compared with the 160 cm bandwidth treatment. However, the kernel number per ear and 1000-seed weight of maize were the highest in the 160 cm bandwidth, increasing by 14.3% and 8.2% respectively compared with the 120 cm bandwidth treatment, and by 28.4% and 6.7% respectively compared with the 80 cm bandwidth treatment (Table 2). The capsule number, seed number per capsule and 1000-seed weight of flax were the highest at a 160 cm bandwidth (except in 2023), increasing by 27.7%, 9.8%, 7.5% and 12.2%, 3.8%, 3.3% respectively compared with the 80 cm and 120 cm bandwidth treatments (Table 3).

### 2.3. Dynamic Dry Matter Accumulation

At the R1 stage, sole-cropped maize exhibited significantly greater dry matter accumulation than intercropped maize, averaging 29.3% higher across years (Figure 3). By the R6 stage, sole cropping still maintained a 16.1% advantage in dry matter accumulation over the 80 cm strip-width treatment. Over the three-year period, within the intercropping system, maize under the 160 cm strip-width treatment showed a marked acceleration in dry matter accumulation starting from the R3 stage. Compared to the 80 cm and 120 cm bandwidth treatments, dry matter accumulation increased by 22.9% and 9.1% at R3 and by 16.8% and 20.8% at R6 in the 160 cm strip-width treatment, respectively.

The dry matter accumulation of sole flax was 14.3% and 5.2% lower than that of the intercropped flax treatments with 160 cm and 120 cm row widths, respectively, at the maturity stage (Figure 3). In the intercropping system, the dry matter accumulation of flax at the anthesis, kernel and maturity stages within the 160 cm bandwidth treatment was 13.5%, 10.6% and 8.7% higher, respectively, than that in the 120 cm bandwidth treatment, and 23.6%, 29.5% and 21.3% higher, respectively, than that in the 80 cm bandwidth treatment. Meanwhile, the 120 cm bandwidth treatment increased the dry matter accumulation of flax by 8.9%, 17.2% and 11.9% at the anthesis, kernel and maturity stages, respectively, compared with the 80 cm bandwidth treatment. This indicates that with the increase in bandwidth in the intercropping system, the dry matter accumulation increases accordingly. The increase in dry matter accumulation of flax is significantly higher than that of maize. The increase in bandwidth of the intercropping system is more beneficial to the growth of flax.

### 2.4. Interspecific Interaction

#### 2.4.1. The Relative Competitiveness of the Intercropped Maize and Flax

There was significant year × treatment interaction affecting the interspecific competition (Figure 4). The relative competitiveness of intercropped maize to flax (A_MF_) across their co-growth periods was evaluated in the maize and flax intercropping system with increased strip width from 2021 to 2023. The A_MF_ was negative during the flax seedling stage (maize V3 stage) in 2022 and 2023, indicating that flax had greater competitiveness than maize. The peak in positivity occurred at the maize V12 stage (the anthesis stage of flax), indicating that maize had a stronger competitive ability than flax. At this period, the highest values were recorded under the 160 cm strip-width treatment, showing mean increases of 22.2% and 46.4% compared to the 120 cm and 80 cm treatments, respectively. At the kernel stage and mature stage of flax (maize VT and R stages), A_MF_ was negative, indicating that flax had a stronger competitive ability than maize. Wider strip widths corresponded to enhanced interspecific competitiveness in flax. The three-year average results showed that during the kernel stage and mature stage, the A_MF_ values under the 160 cm strip-width treatment increased by 121.5% and 73.1%, 99.0% and 95.3% respectively, compared to the 120 cm and 80 cm strip-width treatments. However, there was no significant difference between the 120 cm and 80 cm strip-width treatments.

#### 2.4.2. Relative Crowding Coefficient of Intercropping

The relative crowding coefficient (RCC) for intercropping was employed to measure the growth benefits of intercropping in comparison to the respective sole-cropping system (Figure 4). Over the three-year study period, the RCC value increased gradually from kernel emergence and continued to rise with plant growth, reaching its peak in late August when the intercropped flax approached maturity. The strip width of the intercropping system influenced the RCC values. The mean RCC values in maize–flax intercropping under 160 cm strip-width treatment were 31.1% and 102.3% greater, respectively, than the 120 cm and 80 cm strip-width treatments. At the maturity stage of flax, the average RCC showed no significant difference between the 160 cm and 120 cm strip-width treatments, but was on average 197.4% higher than that of the 80 cm strip-width treatment. The results indicate that increasing the strip width contributes to enhancing the RCC, thereby providing a favorable basis for improved growth.

#### 2.4.3. Recovery Effect in Intercropping System

After the harvest of flax, the effects of different intercropping strip widths on the recovery effect (RE) were significant. Except for the RE of the 80 cm strip-width treatment in 2023 being less than 1, the R values of all other treatments were greater than 1 (Figure 5). The results of three consecutive years of experiments indicated that the 160 cm strip-width treatment had a distinct advantage, with its R value increasing by 18.5% and 44.2% respectively compared to the 120 cm and 80 cm strip-width treatments. Meanwhile, the RE increased significantly by 21.8% under the 120 cm bandwidth treatment compared to the 80 cm bandwidth.

### 2.5. Correlation Between the Grain Yields and Interspecific Competition

Correlation analysis demonstrated that the A_MF_ at VT stage of maize was significantly positively correlated with capsule number of flax (Figure 6). At the V3 and V12 stages of maize, the A_MF_ exhibited an extremely significant negative correlation with flax yield. Meanwhile, the A_MF_ during at V3 stage was negatively correlated with the grain yield of maize. The number of ears per maize plant exhibits a significant negative correlation with both grain yield of maize and total system yield. The 1000-seed weight of flax shows a significant negative correlation with the total system yield. These findings suggest that strategic manipulation of competition during the maize V3 and V12 stages can optimize yield in this maize–flax intercropping system.

## 3. Discussion

### 3.1. Intercropping Advantages

Rational spatial configuration and strip-width regulation is critical to tapping the yield potential of strip intercropping systems via ecological niche differentiation and resource complementarity [25,26,27]. Consistent with the present findings, optimized row and strip spatial arrangements can effectively alleviate interspecific competition and improve overall resource use efficiency, thereby generating prominent intercropping yield advantages [28,29]. The LER is a reliable quantitative index for evaluating the resource utilization superiority of intercropping systems, with an LER value above 1.0 indicating a pronounced yield benefit relative to monocropping [30,31,32]. In this study, the average LER of the maize–flax strip intercropping system reached 1.66, fully verifying that strip intercropping substantially improves the utilization efficiency of limited farmland and light resources compared with sole cropping. Notably, the 160 cm strip-width treatment (M4F8) yielded the maximum system LER, which was 24.4% higher on average than that of other strip-width settings (M2F4 and M3F6), demonstrating that this optimal spatial configuration maximizes the intercropping superiority of the system. Further analysis of the partial land equivalent ratio (PLER) indicated that the elevated system LER under the 160 cm strip width was primarily attributed to the significantly improved PLER of maize, while the PLER of flax remained stable across different strip-width treatments. Across all planting configurations, maize exhibited a consistently higher PLER than flax, confirming its dominant competitive position in the intercropping system. This competitive predominance may be mainly attributed to the superior canopy structure of maize, which enables more effective interception and utilization of aboveground photosynthetically active radiation, thereby contributing to asymmetric light competition and resource partitioning within the intercropping system [33,34]. These results further illustrate that reasonable strip-width regulation optimizes canopy light distribution and interspecific niche coordination, which is the key mechanism for improving yield performance and resource utilization efficiency of semi-arid rainfed agricultural area maize/flax intercropping systems.

From a yield perspective, although flax yield in the maize–flax intercropping system was partially reduced relative to sole flax, the total system yield (combining both maize and flax grain yield) in intercropping was markedly higher than that under sole-cropping systems. This indicates that rationally designed strip-intercropping configurations can substantially raise overall land productivity. The elevated total system yield reflects improved land-resource use efficiency achieved by maize–flax intercropping. By combining tall-growing C_4_ maize and short-stature C_3_ flax, the intercropping system taps into the productive capacity of farmland by taking advantage of niche complementarity between the two crop species [35,36]. This strategy delivers greater yield output per unit land area compared with sole cropping. Notably, despite inter-annual climatic fluctuation across experimental years, maize–flax strip intercropping achieved higher total productivity and favorable land equivalent ratios. These results further support the practicability and superiority of the maize–flax intercropping model for local agricultural production, offering farmers options for stable and enhanced land productivity.

The increase in system-level harvest index (HI) was closely associated with total biomass accumulation of the intercropping system [37]. Among component species, maize was the primary driver for this variation. Owing to its higher individual productivity, maize contributes the largest proportion to the total biomass of the intercropping community [38]. Consequently, changes in maize biomass largely determine the total biomass of the whole system, and further regulate the variation of system HI. This indicates that the performance of maize plays a decisive role in the harvest index performance of mixed intercropping systems. Future intercropping practice should pay more attention to optimizing maize growth to improve the resource-use efficiency of the whole system.

### 3.2. Strip Width Optimizes Yield by Yield Components in Maize–Flax Intercropping Systems

The yield of cereal crops is determined by the number of ears per unit area, the kernel number per ear and the 1000-seed weight [39]. The coordination of the three is the basis for high yield. Intercropping, through a reasonable spatial structure, alleviates the contradiction between individuals and the group, promoting the coordinated development of the number of ears, the kernel per ear and the 1000-seed weight [40,41,42]. This study found that the intercropping system has a positive regulatory effect on the yield components of maize and flax. The number of ears per area of maize significantly increased by 27.8% under the intercropping condition, which might be due to the improvement of the field microenvironment by the intercropping system, enhancing the competitive advantage of corn in the early growth stage, and thereby promoting the effective establishment of its population [43,44]. No significant differences were found in kernel number per ear or 1000-kernel weight between systems, indicating that yield in intercropped maize is driven by higher ear density, not individual ear productivity. In contrast, flax showed greater responsiveness, with capsule, seeds per capsule, and 1000-seed weight increasing by 51.4%, 20.6%, and 13.6%, respectively. These results suggest that flax improves development by using light, water, and nutrients more efficiently than maize, gaining advantages across multiple yield traits.

Strip width, a key spatial factor in intercropping, strongly influences crop yield. This study shows that maize and flax respond differently to strip width, reflecting trade-offs between competition and resource use. For maize, a narrow strip width (80 cm) maximizes ear number, likely due to higher plant density and greater competitive advantage [45]. In contrast, wider bandwidth (160 cm) increases kernel number per ear and 1000-seed weight in intercropped maize, indicating improved resource availability—such as light, water, and nutrients—for individual plants, supporting better grain filling. Thus, maize yield under the 80 cm intercropping strip width relies on ear number, while that under the 160 cm strip width relies on improved kernel number per ear and 1000-kernel weight in intercropping system. Unlike maize, all the yield components of intercropped flax reached their peaks at the 160 cm strip-width treatment. This clearly indicates that a wider strip width effectively alleviates the shading and competition pressure from maize on flax, creating a more relaxed and favorable growth space for flax, allowing it to fully express its yield potential [46].

### 3.3. Correlation Between Interspecific Relationships and Strip Width in Maize–Flax Intercropping Systems

This study demonstrates that strip width is a critical factor regulating interspecific competition and productivity in maize–flax intercropping systems. Consistent with previous research, the competitiveness of crops in intercropping systems varies dynamically across growth stages [47]. Specifically, this study observed that flax exhibited a competitive advantage over maize during its seedling and maturity stages, whereas maize reached its peak competitive ability at the V12 stage, coinciding with the flowering stage of flax. These competitive dynamics are closely associated with the resource capture patterns and canopy architecture of the crops, supporting the manifestation of the “niche complementarity” theory in the temporal dimension [48]. Notably, the 160 cm strip-width treatment significantly alleviated the intensity of interspecific competition while enhancing the RCC and RE of the system. These findings are consistent with previous studies, which suggest that appropriately increasing row spacing or strip width can optimize resource-use efficiency [49]. Furthermore, correlation analysis elucidated the underlying mechanisms linking competition and yield formation: the competitive ability of maize at the VT stage was positively correlated with the capsule number of flax, whereas excessively strong competition during the early stage (V3) negatively impacted maize’s own grain yield. This suggests a significant resource trade-off between competitive traits and reproductive growth in intercropped species. In conclusion, optimizing strip width effectively balances interspecific competition through spatiotemporal niche differentiation, thereby providing a practical management strategy for developing highly productive and efficient intercropping systems.

Limitations of this study should be noted when interpreting our findings. First, the field experiment was carried out at a single experimental site over limited growing seasons. Environmental conditions such as temperature and precipitation vary across years and locations; therefore, our conclusions should be extrapolated to other agro-ecological zones with caution. Second, we mainly focused on yield, biomass and competitive indices in the present work. Further measurements including soil nutrient dynamics, root distribution and rhizosphere microbial properties were not fully considered, which may help to further unravel interspecific interaction mechanisms. Third, this study only tested several strip-width treatments. Different planting densities, fertilization levels and cultivar types may modify interspecific competition and resource complementarity. Future multi-site and multi-year field trials combined with multi-dimensional indicators are required to validate and extend our results.

## 4. Materials and Methods

### 4.1. Experimental Site

The field experiments were carried out at the Xizhai Experiment Station of Dingxi Academy Agricultural Science in Dingxi (34°26′ N, 103°52′ E), Gansu, China from 2021 to 2023. Dingxi is located at the intersection of the Loess Plateau, the Tibetan Plateau and the Western Qinling Mountains in northwestern China, which are mainly characterized by a semi-arid rainfed agricultural area. Total rainfall during the growing season (April to September) was 286.0 mm in 2021, 246.8 mm in 2022, and 305.8 mm in 2023 (Figure 7). The annual mean temperature is 6.3 degrees Celsius, and there are typically 2453 h of sunshine per year. Soil texture is cultivated loessial soils with a bulk density of 1.43 g cm^−3^, 17.51 g kg^−1^ of soil organic matter, 1.05 g kg^−1^ of total N, 26.43 mg kg^−1^ of available P and 108.32 mg kg^−1^ of available K in the top 30 cm of the soil profile. The experimental field was plowed after the previous growing season. Before sowing in the following year, shallow tillage followed by harrowing and leveling was conducted to prepare the soil for sowing. A pre-emergence herbicide was applied before sowing for weed control. During the growing season, pests and diseases were monitored, and appropriate control measures were implemented, when necessary, in accordance with local agricultural practices.

### 4.2. Experimental Design

An experiment was conducted as a randomized complete block design with three replicates. Three strip-width treatments were designed in the intercropping system, consisting of three intercropping combinations: 2 rows of maize intercropped with 4 rows of flax (M2F4, strip width of 80 cm:80 cm), 3 rows of maize intercropped with 6 rows of flax (M3F6, strip width of 120 cm:120 cm), and 4 rows of maize intercropped with 8 rows of flax (M4F8, strip width of 160 cm:160 cm). Maize and flax had a 50% land proportion in each of the intercrop treatments. Two pure stands acted as reference: sole maize (SM) and sole flax (SF). Thus, there were a total of 5 treatments with three replicates, constituting 15 plots in this study.

Maize Xian-yu 335 and flax Longya 11 are widely planted in Northwest China, and they were planted in a maize/flax intercropping system. Maize was planted on 28th April 2021, 1st May 2022, and 28th April 2023 and harvested on 29th September 2021, 2nd October 2022, and 10th October 2023. Flax was planted on 11th April 2021, 27th April 2022, and 12th April 2023 and harvested on 7th August 2021, 10th August 2022, and 12th August 2023.

The plot sizes were 4.8 m × 1.6 m for M2F4, 7.2 m × 1.6 m for M3F6, 9.6 m × 1.6 m for M4F8, and 5.0 m × 2.0 m for both sole M and sole F treatments. Row space of maize and flax was 40 cm and 20 cm, respectively, and adjacent rows are spaced 30 cm apart (Figure 8). Each intercropping plot contained three complete intercropping strips, each consisting of three maize strips and three oilseed flax strips, to ensure representative sampling of soil and plants. The planting densities for intercropped maize and flax were 6.0 × 10^4^ plant hm^−2^ and 7.5 × 10^6^ plant hm^−2^, respectively. The intercrops were planted at the same density as the sole crops. For maize, the total N application was 250 kg ha^−1^, with 60% applied as base fertilizer along with the full P_2_O_5_ rate (125 kg ha^−1^), and the remaining 40% of N was supplied as a topdressing at the jointing stage. Only basal fertilizer was applied for flax (120 kg ha^−1^ N and 75 kg ha^−1^ P_2_O_5_). The study region was a typical semi-arid rainfed agricultural area and does not require additional irrigation. The phenological stages of maize and flax observed at the Dingxi Experimental Station across the 2021, 2022, and 2023 growing seasons are presented in Table 4. To minimize border effects and protect the experimental plots from external disturbances, 1.0 m wide border strips were established around the experimental area and planted with flax. These border rows were managed in the same manner as the experimental plots.

### 4.3. Measurements

#### 4.3.1. Yield and Yield Components

All experimental plots were harvested by hand when the crops reached full maturity status. The grains were determined based on air-drying to achieve a moisture content of approximately 14% for cleaned crops of different treatments in the sole crops and intercrops individually. For the intercropped plots, one pair of flax and maize strips was designated for destructive sampling to determine aboveground biomass at different growth stages, whereas the remaining two pairs of flax and maize strips were retained for grain yield determination at physiological maturity. In the sole-cropped plots, half of the plot area was designated for destructive sampling to determine aboveground biomass at different growth stages, while the remaining half was retained for grain yield determination at physiological maturity. Total system yield was calculated as below:

(1) $$Y_{T}=0.5Y_{M}+0.5Y_{F}$$

In maize, the number of ears was counted and weighed, with 10 randomly selected ears further analyzed to determine the number of kernels per ear and the weight of 1000 kernels. For flax, measurements included the capsule number, the number of seeds per capsule, and the weight of 1000 seeds, assessed across all plants within the sampling area.

#### 4.3.2. Land Equivalent Ratio

The land equivalent ratio (LER) is a metric used to assess the productivity and efficiency of intercropping systems and evaluate the utilization of land resources [50]. The partial land equivalent ratio (pLER) for flax (pLER_F_) and maize (pLER_M_) was calculated to assess the contribution of each crop to the overall productivity of the intercropping system. LER assumes consistent management between monoculture and intercropping. While LER > 1 indicates a land-use advantage, it does not distinguish whether this advantage arises from resource complementarity or reduced competition. LERs are calculated using the following equations:

(2) $${pLER}_{F}=\frac{Y_{IF}}{Y_{SF}}$$

(3) $${pLER}_{M}=\frac{Y_{IM}}{Y_{SM}}$$

(4) $$LER={pLER}_{F}+{pLER}_{M}$$

where Y_IF_ and Y_IM_ are the yields of flax and maize in the intercropping system, respectively; Y_SM_ and Y_SF_ are the yields of oilseed flax and maize in sole crops, respectively.

#### 4.3.3. Dry Matter and Harvest Index

The aboveground dry matter of maize was quantified on V3, V6, V12, VT, R1, R3 and R6 stages, and flax was quantified on seeding, budding, anthesis, kernel, and maturity stages across all treatments. Within each plot, 10 flax plants and 3 maize plants from a sub-sampling area were randomly chosen to assess the final dry matter of the crops. All aboveground samples were initially oven-dried at 105 °C for one hour to inactivate enzymes and subsequently dried at 85 °C for 48 h until a constant weight was achieved. The final dry matter was weighted on an electronic balance. The harvest index (HI) was determined by dividing the grain yield by the final dry matter for each plot.

#### 4.3.4. The Competitiveness of Intercropped Maize to Flax (A_MF_)

The A_MF_ was quantified as follows [51]:

(5) $$A_{MF}=\frac{Y_{IM}}{Y_{SM}P_{M}}-\frac{Y_{IF}}{Y_{SF}P_{F}}$$

where P_M_ and P_F_ are the proportions of sowing area for intercropped maize and intercropped flax in the intercropping system (in this research, P_M_ = P_F_ = 0.5); Y_IM_ and Y_IF_ are the dry matter accumulation at the corresponding growth stage of the intercropped maize and intercropped flax, respectively. An A_MF_ value greater than zero indicates that intercropped maize exhibits higher competitive ability relative to intercropped flax, thereby establishing maize as the dominant species in the maize/flax intercropping system; conversely, when the A_MF_ is less than zero, flax becomes the dominant component.

The relative crowding coefficient (RCC). The RCC is calculated using the following equations:

(6) $$RCC={RCC}_{maize}\times {RCC}_{flax}$$

(7) $${RCC}_{maize}=\frac{Y_{IM}P_{F}}{\left(Y_{SM}-Y_{IM}\right)P_{M}}$$

(8) $${RCC}_{flax}=\frac{Y_{IF}P_{M}}{\left(Y_{SF}-Y_{IF}\right)P_{F}}$$

where RCC, RCC_maize_, RCC_flax_ are the relative crowding coefficients of the intercropping system, intercropped maize, and intercropped flax, respectively. Other variables are as described in Equation (5) above. In the above three equations, RCC > 1 indicates that the intercropping pattern has a yield advantage in comparison with the sole pattern, RCC = 1 indicates no yield advantage of intercropping, and RCC < 1 indicates an intercropping disadvantage over the sole system.

The recovery effect (RE) indicates the degree of recovery of production performance of maize after flax harvest in the intercropping system. The calculation formula is as follows:

(9) $$RE=\frac{D_{IM2}-D_{IM1}}{D_{SM2}-D_{SM1}}$$

D_IM2_ and D_SM2_ represent the dry matter accumulation (DMA) of intercropped and sole maize at maturity, respectively, while D_IM1_ and D_SM1_ represent the DMA at the R1 stage of maize (after flax harvest). A value of RE higher than 1.0 indicates intercropped maize has compensation effect after accompanying flax harvest, and vice versa.

### 4.4. Statistical Analysis

The data were processed using statistical analysis software (SPSS version 17.0, SPSS Inst. Ltd., USA). Treatment effects were examined by applying the LSD multiple-range test at a significance level of 0.05. Due to significant year × treatment interactions observed for most of the assessed variables, the effect of treatment was analyzed separately for each year. Correlation analyses between intercropping-advantage-related factors and crop yield were performed using the vegan package in R (version 4.2.1).

## 5. Conclusions

In a maize–flax intercropping system, the 160 cm strip-width treatment demonstrated system optimization through multiple synergistic mechanisms. This configuration significantly enhanced both grain and biomass yields, with the LER increasing progressively with wider strips, particularly driven by a pronounced improvement in the PLER of maize. Yield component analysis revealed that the increase in maize yield was attributed to coordinated improvements in kernel number per ear and 1000-kernel weight, while flax yield enhancement resulted from simultaneous increases in capsule number per plant, seeds per capsule, and 1000-seed weight. These yield advantages were closely associated with significantly enhanced post-anthesis dry matter accumulation in flax, coupled with strengthened competitive ability during its late growth stages under the 160 cm strip width. From a system perspective, widening the strip spacing significantly elevated the RCC and RE, indicating enhanced overall growth dominance and more favorable recovery conditions for subsequent maize. Importantly, interspecific competitiveness during the maize V3 and V12 stages showed significant negative correlations with flax yield, providing critical insights for optimizing intercropping productivity through strategic manipulation of interspecific competition.

## Figures and Tables

**Figure 1:**
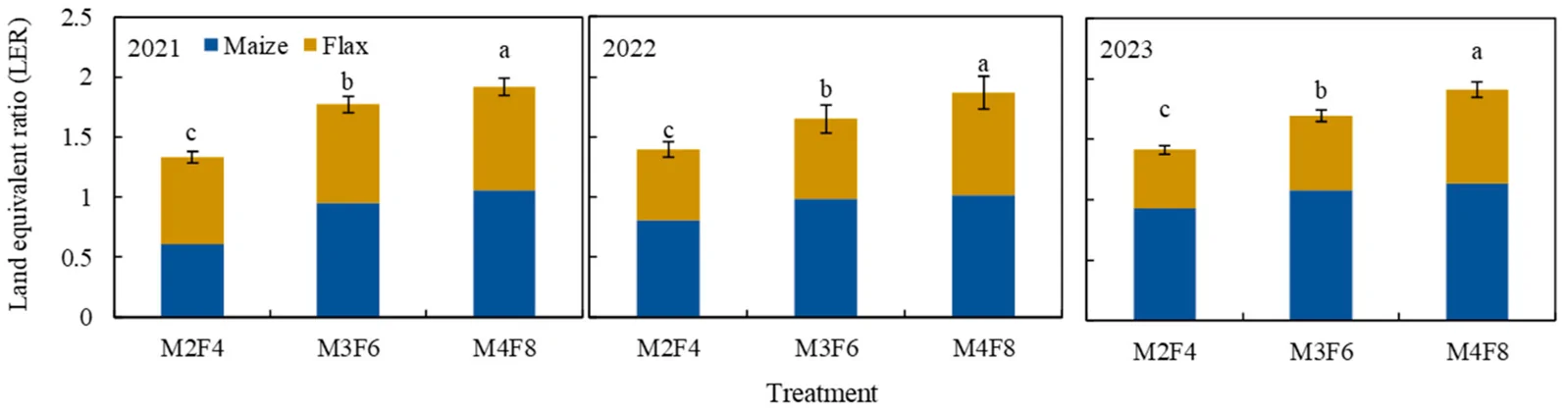
Land equivalent ratio (LER) as affected by different strip widths in intercropping systems in 2021 to 2023. M2F4: two rows of maize and four rows of flax, with a strip-width ratio of 80 cm:80 cm. M3F6: three rows of maize and six rows of flax, with a strip-width ratio of 120 cm:120 cm. M4F8: four rows of maize and eight rows of flax, with a strip-width ratio of 160 cm:160 cm. The error bars indicate the SEM (n = 3). No common letter above bars indicates significant differences at the *p* < 0.05 probability level.

**Figure 2:**
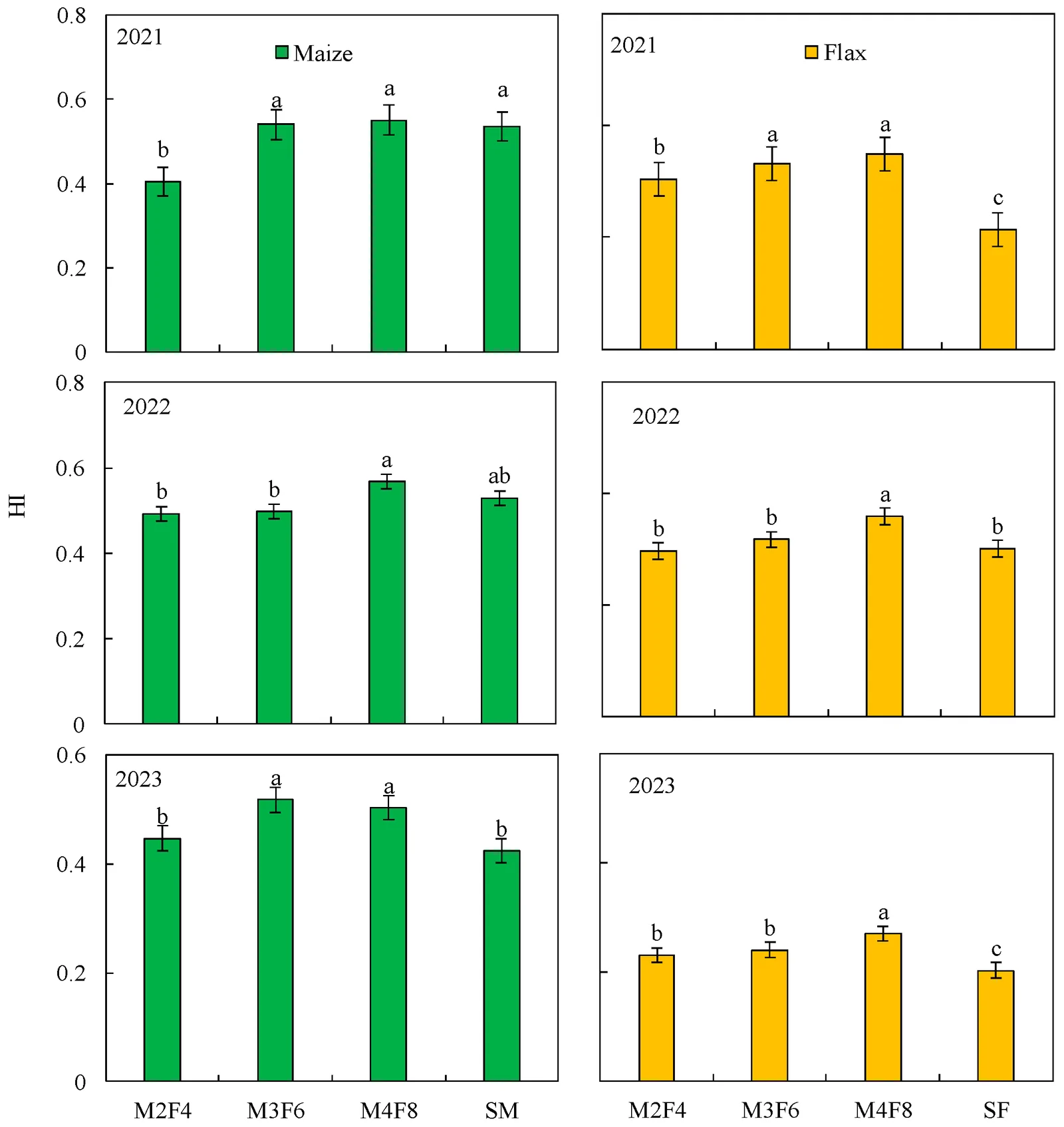
Harvest index (HI) as affected by different strip widths in intercropping systems in 2021 to 2023. M2F4: two rows of maize and four rows of flax, with a strip-width ratio of 80 cm:80 cm. M3F6: three rows of maize and six rows of flax, with a strip-width ratio of 120 cm:120 cm. M4F8: four rows of maize and eight rows of flax, with a strip-width ratio of 160 cm:160 cm. SM: monocropped maize. SF: monocropped flax. The error bars indicate the SEM (n = 3). No common letter above bars indicates significant differences at the *p* < 0.05 probability level.

**Figure 3:**
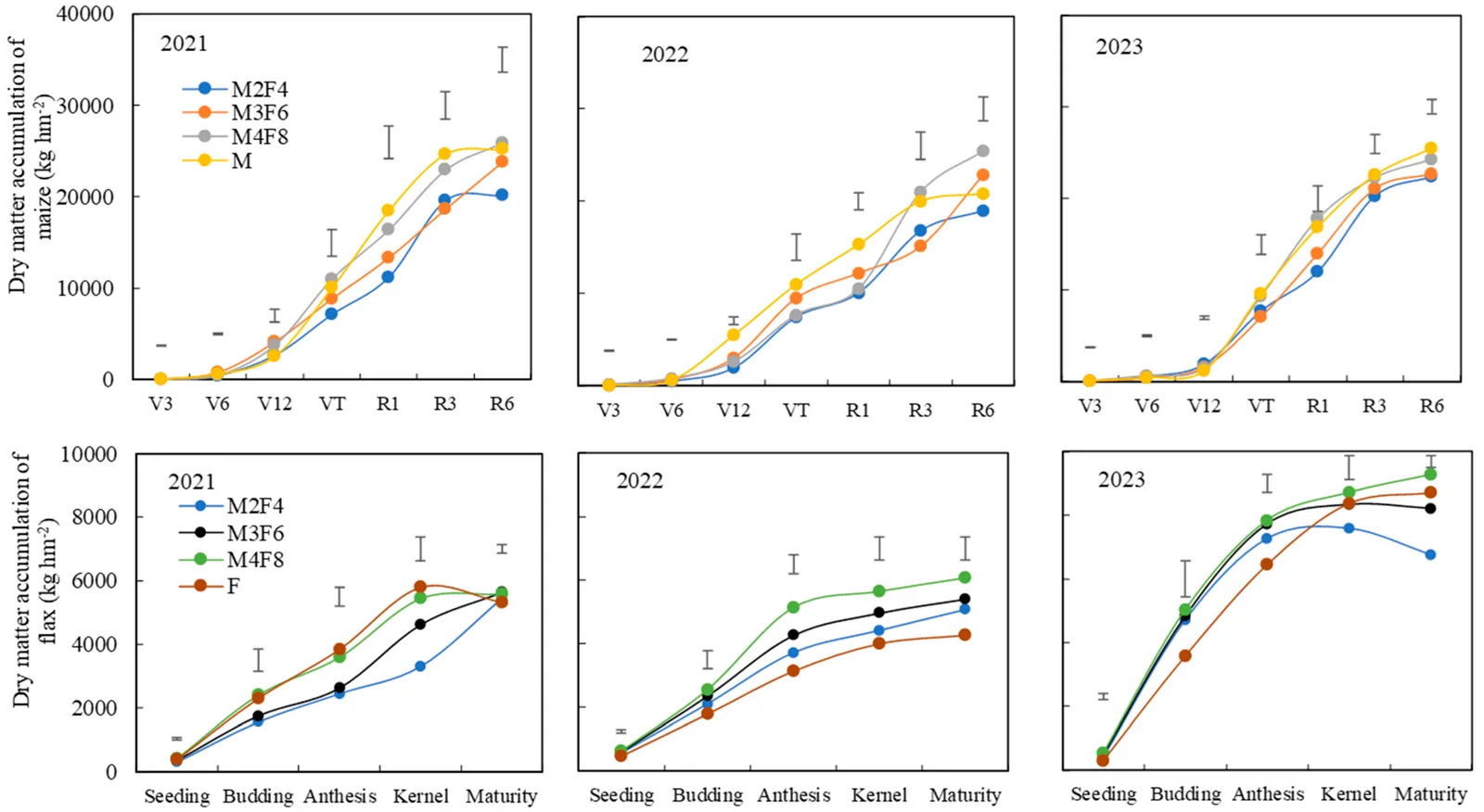
The dry matter accumulation of maize and flax in 2021 to 2023. M2F4: 2 rows of maize and 4 rows of flax, with a strip-width ratio of 80 cm:80 cm. M3F6: 3 rows of maize and 6 rows of flax, with a strip-width ratio of 120 cm:120 cm. M4F8: 4 rows of maize and 8 rows of flax, with a strip-width ratio of 160 cm:160 cm. M: monocropped maize. F: monocropped flax. The vertical bars represent the LSD at *p* =0.05 among various treatments within a measurement date.

**Figure 4:**
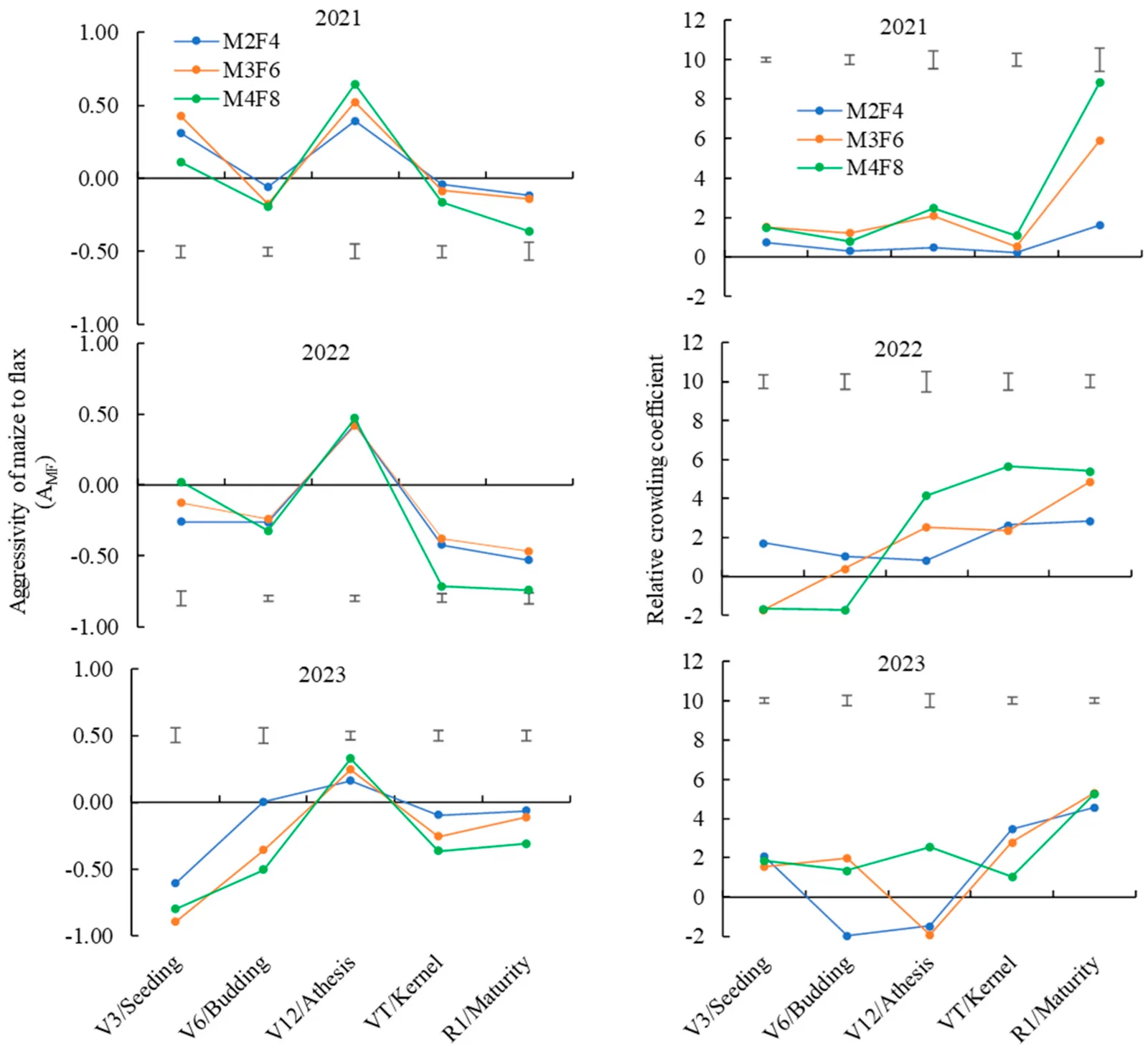
The aggressivity (maize to flax, A_MF_) and relative crowding coefficient (RCC) maize–flax intercropping system in 2021 to 2023. M2F4: two rows of maize and four rows of flax, with a strip-width ratio of 80 cm:80 cm. M3F6: three rows of maize and six rows of flax, with a strip-width ratio of 120 cm:120 cm. M4F8: four rows of maize and eight rows of flax, with a strip-width ratio of 160 cm:160 cm. The error bars indicate the SEM (n = 3).

**Figure 5:**
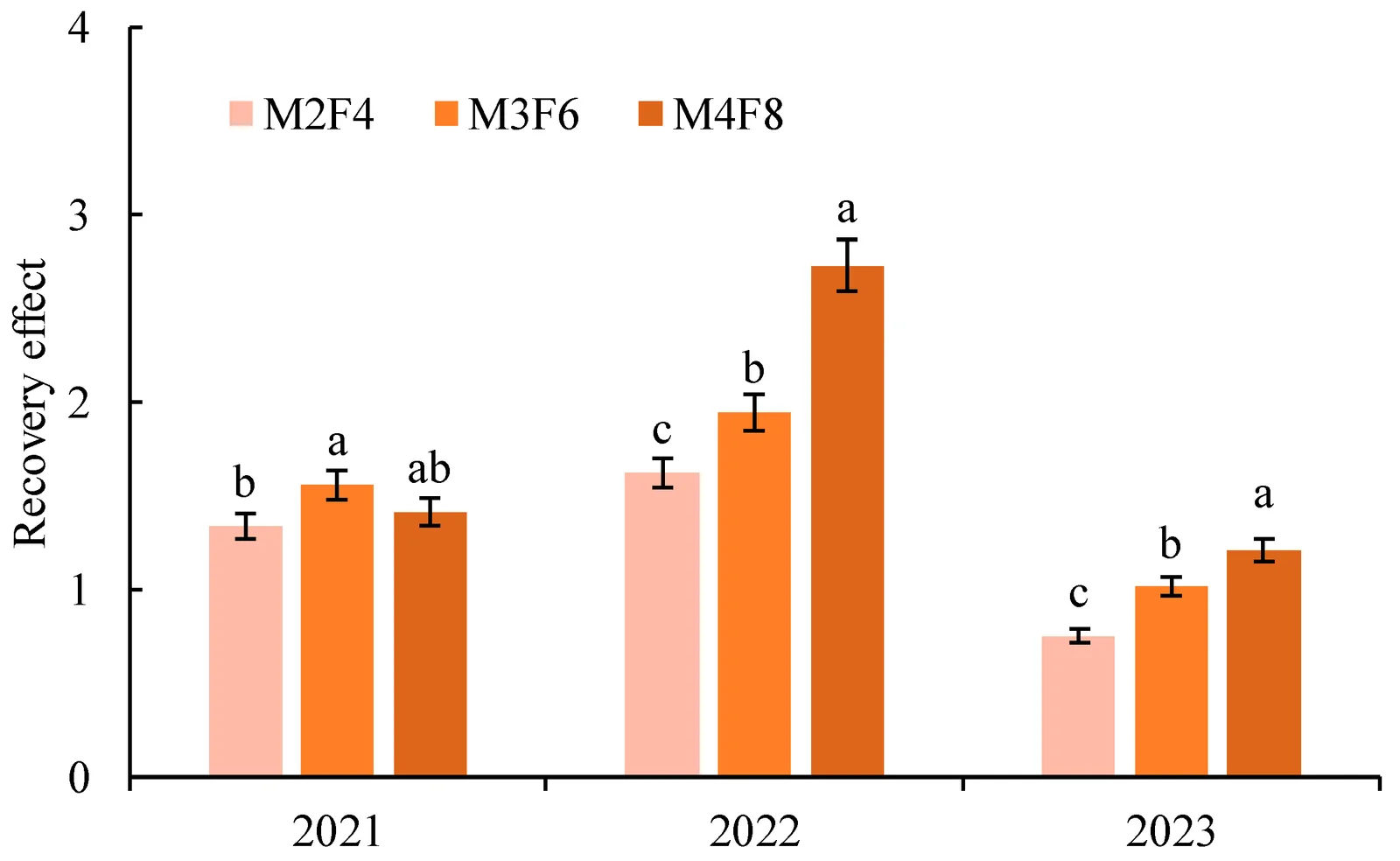
The recovery effect of maize in intercropping systems in 2021 to 2023. M2F4: two rows of maize and four rows of flax, with a strip-width ratio of 80 cm:80 cm. M3F6: three rows of maize and six rows of flax, with a strip-width ratio of 120 cm:120 cm. M4F8: four rows of maize and eight rows of flax, with a strip-width ratio of 160 cm:160 cm. Different small letters indicate significant differences under the same treatment at *p* < 0.05 as determined by the LSD test. The error bars indicate the SEM (n = 3).

**Figure 6:**
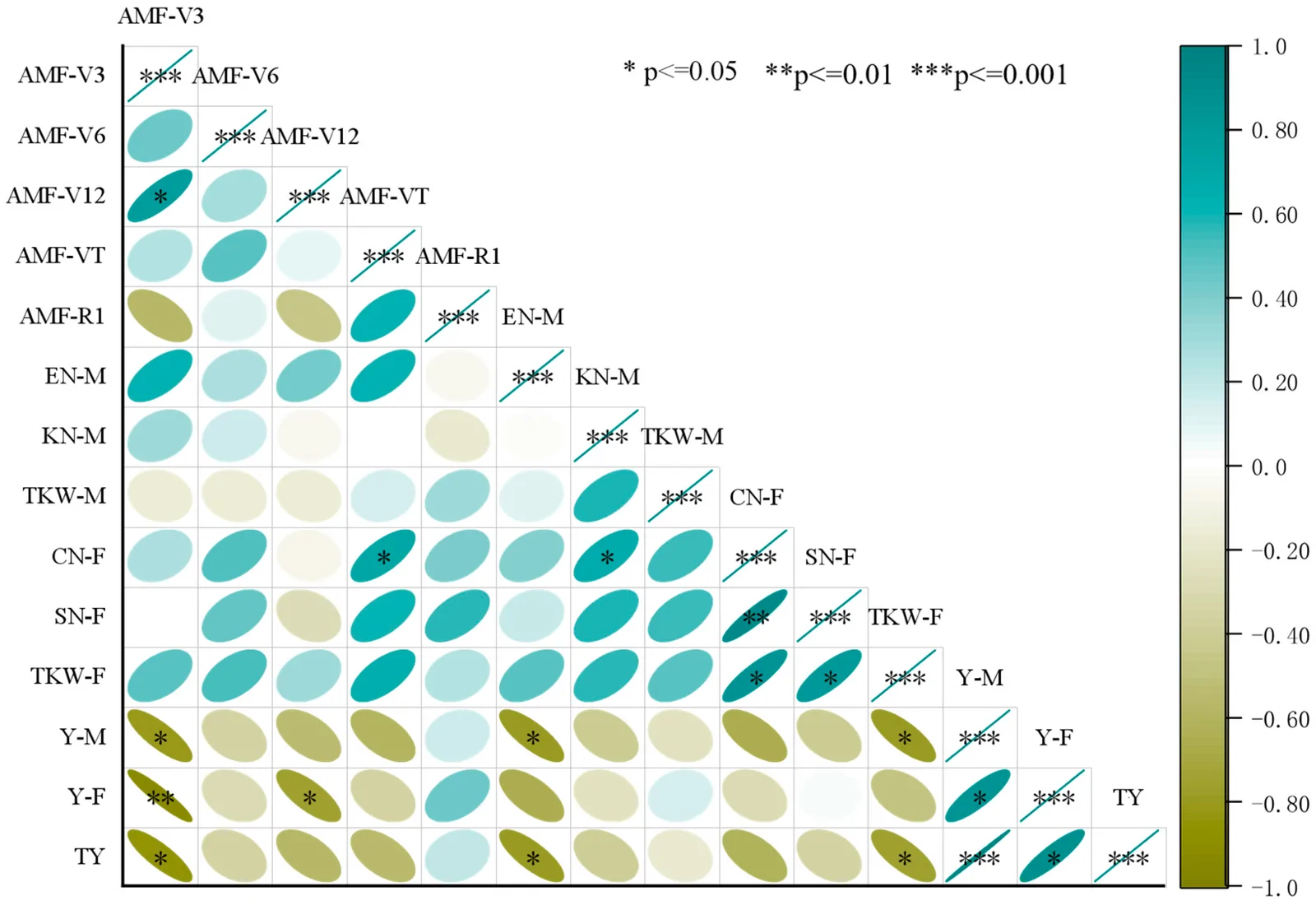
Correlations among yield, yield components and interspecific competition in the intercropping system. Data were pooled across 2021–2023, with a sample size of n = 27 for each correlation. Although significant year-by-treatment interactions existed (Table 1, Table 2 and Table 3), correlation trends were consistent across individual years; thus, pooling was justified to explore general variable associations. The size and color of the blocks in the figure indicate the strength of the positive and negative correlations among various factors. TY, Y-F and Y-M represent total system yield of the intercropping system, the yield of flax in intercropping and the yield of maize in intercropping, respectively. CN-F, SN-F and TKW-F represent capsule number, seed number per capsule and 1000-seed weight in intercropped flax, respectively. EN-M, KN-M and TKW-M represent ear number, kernel number per ear and 1000-seed weight in intercropped maize, respectively. AMF-V3, AMF-V6, AMF-V12, AMF-VT and AMF-R1 represent competitiveness of intercropped maize to flax in the V3, V6, V12, VT and R1 maize stage, respectively. * *p* < 0.05, ** *p* < 0.01, *** *p* < 0.001. n = 27. This correlation analysis chart is a statistical analysis based on data collected from all treatments across 2021–2023.

**Figure 7:**
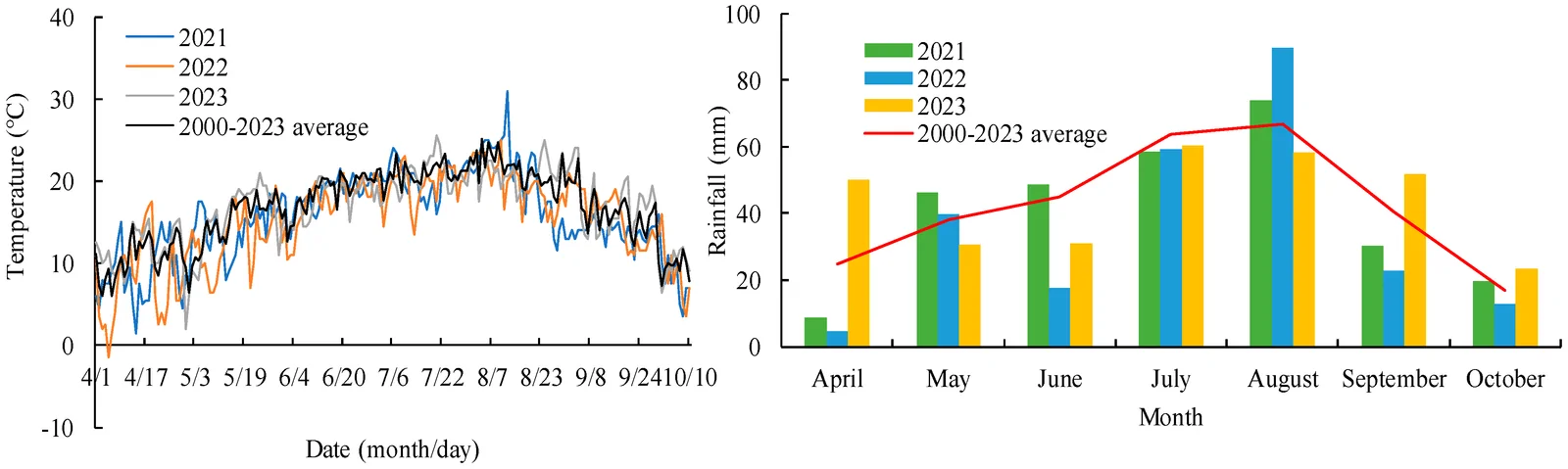
Rainfall and air temperature during maize and flax growing seasons (from 1 April to 10 October) in 2021, 2022, and 2023 in comparison with the long-term (2000–2023) average from the National Meteorological Information Center, at the Dingxi Experimental Station near, Gansu, China.

**Figure 8:**
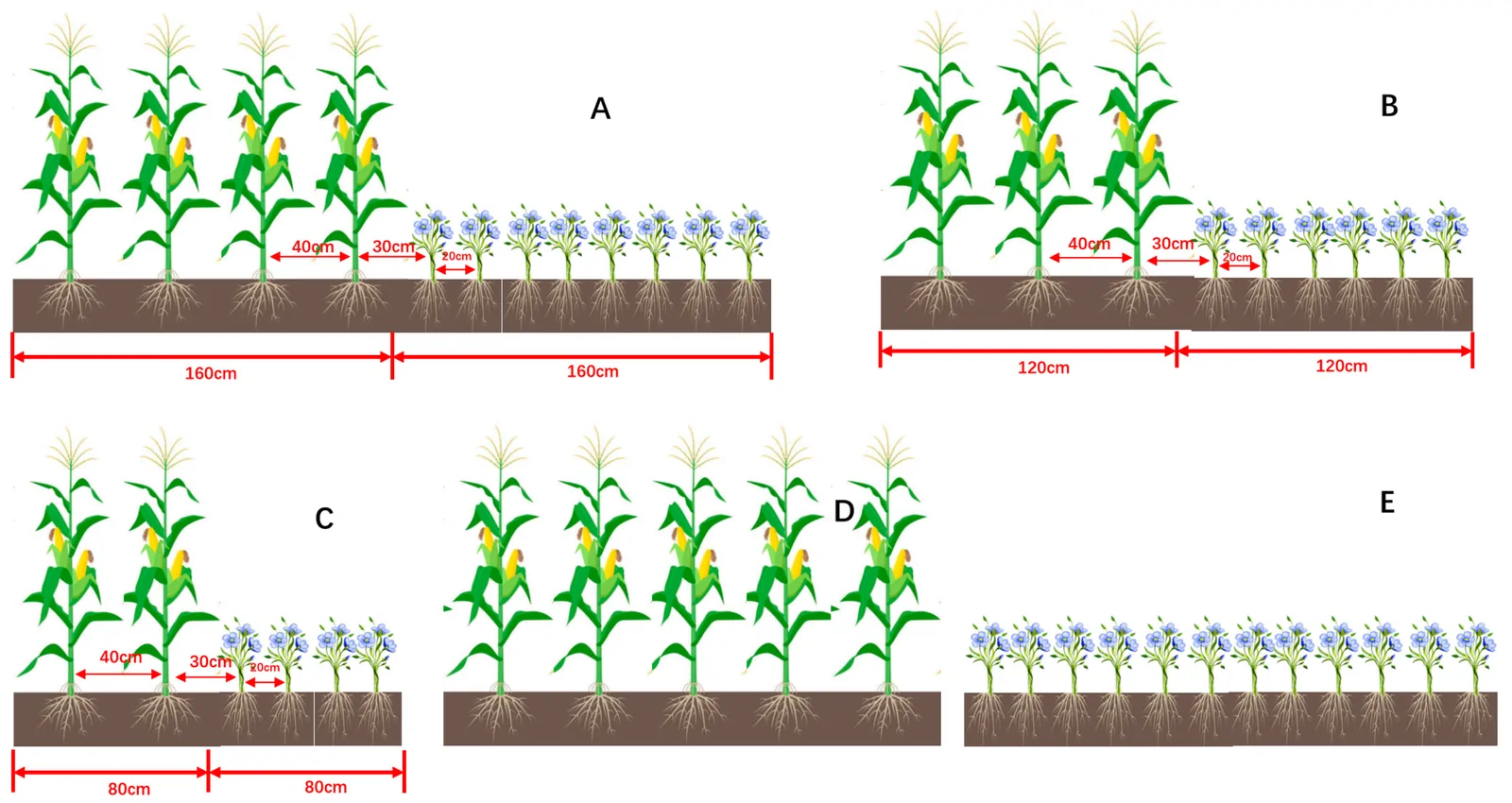
Layout of maize/flax intercropping in field experiment. Three strip-width treatments were designed in the intercropping system: (**A**): 4 rows of maize intercropped with 8 rows of flax (M4F8, strip width of 160 cm:160 cm), (**B**): 3 rows of maize intercropped with 6 rows of flax (M3F6, strip width of 120 cm:120 cm) and (**C**): 2 rows of maize intercropped with 4 rows of flax (M2F4, strip width of 80 cm:80 cm). (**D**) and (**E**) represent monoculture maize and flax, respectively.

**Table 1:** Grain yields and biological yield of the intercrops in 2021, 2022 and 2023.

Year	Treatment	Maize Grain Yield		Flax Seed Yield		Total System Yield		Maize Biological Yield		Flax Biological Yield		Total Biological Yield	
2021	M2F4	8150.9	d	825.6	c	4488.2	d	20,174.8	c	2714.2	b	11,444.4	d
	M3F6	12,835.1	c	934.1	bc	6884.6	c	23,769.1	b	2818.4	b	13,293.8	c
	M4F8	14,262.7	a	973.7	b	7618.2	b	25,900.2	a	2786.4	b	14,343.3	b
	SM	13,488.7	b			13,488.7	a	25,173.4	a			25,173.4	a
	SF			1137.6	a	1137.6	e			5317.7	a	5317.7	e
2022	M2F4	9369.5	b	751.8	d	5060.6	c	18,971.9	d	2530.1	d	10,751.0	c
	M3F6	11,375.7	a	856.1	c	6115.9	b	22,896.0	b	2694.9	c	12,795.5	b
	M4F8	11,783.5	a	1089.6	b	6436.6	b	20,795.9	c	3031.3	b	11,913.6	b
	SM	11,587.9	a			11,587.9	a	25,469.2	a			25,469.2	a
	SF			1283.1	a	1283.1	d			4253.0	a	4253.0	d
2023	M2F4	10,052.9	c	779.8	d	5416.4	d	22,416.7	b	3380.2	b	12,898.4	d
	M3F6	11,693.1	b	983.1	c	6338.1	c	22,682.6	b	4108.0	b	13,395.3	c
	M4F8	12,169.3	a	1252.2	b	6710.8	b	24,267.7	a	4642.6	b	14,455.1	b
	SM	10,780.4	c			10,780.4	a	25,503.2	a			25,503.2	a
	SF			1614.4	a	1614.4	e			8718.9	a	8718.9	e
Significant													
Year		0.003 **		0.000 **		0.000 ***		0.000 **		0.000 **		0.000 **	
Treatment		0.000 **		0.000 **		0.000 ***		0.007 **		0.000 **		0.000 **	
Year × Treatment		0.001 **		0.000 **		0.000 ***		0.001 **		0.024 **		0.000 **	

Note: The unit of yield for the single-cropped and intercropped crops is kg ha^−1^. M2F4: two rows of maize and four rows of flax, with a strip-width ratio of 80 cm:80 cm. M3F6: three rows of maize and six rows of flax, with a strip-width ratio of 120 cm:120 cm. M4F8: four rows of maize and eight rows of flax, with a strip-width ratio of 160 cm:160 cm. SM: monocropped maize. SF: monocropped flax. Different small letters indicate significant differences under the same treatment at *p* < 0.05, as determined by the LSD test; ** and *** indicate statistical significance at the 0.01 and 0.001 levels, respectively.

**Table 2:** Yield components of maize under intercropping and monoculture treatments in 2021 to 2023.

Year	Treatment	Average Number Ears (m^−2^)		Kernel Number Per Ear		1000-Kernel Weight (g)	
2021	M2F4	13.0	a	428.0	d	304.2	b
	M3F6	10.3	b	468.0	c	302.9	b
	M4F8	11.7	ab	506.7	a	337.6	a
	SM	8.0	c	488.0	b	306.2	b
2022	M2F4	10.0	a	408.0	b	309.3	b
	M3F6	9.0	ab	449.3	ab	300.0	c
	M4F8	9.7	a	502.7	a	320.3	a
	SM	8.7	b	473.3	ab	306.0	bc
2023	M2F4	10.3	a	347.1	b	310.3	b
	M3F6	8.7	b	411.2	ab	308.2	c
	M4F8	9.3	ab	510.0	a	328.1	a
	SM	7.3	c	388.4	ab	315.0	b
Significant							
Year		0.003 **		0.024 *		0.003 **	
Treatment		0.000 **		0.018 *		0.000 **	
Year × Treatment		0.322		0.058		0.001 **	

Note: M2F4: two rows of maize and four rows of flax, with a strip-width ratio of 80 cm:80 cm. M3F6: three rows of maize and six rows of flax, with a strip-width ratio of 120 cm:120 cm. M4F8: four rows of maize and eight rows of flax, with a strip-width ratio of 160 cm:160 cm. SM: monocropped maize. SF: monocropped flax. Different small letters indicate significant differences under the same treatment at *p* < 0.05 as determined by the LSD test; * significant at *p* < 0.05. **, significant at *p* < 0.001.

**Table 3:** Yield components of flax under intercropping and monoculture treatments in 2021 to 2023.

Year	Treatment	Capsule Number (Plant^−1^)		Seed Number Per Capsule		1000-Seed Weight (g)	
2021	M2F4	20.5	c	8.3	c	7.3	c
	M3F6	23.5	b	8.8	b	7.6	b
	M4F8	26.3	a	8.9	a	7.8	a
	SF	10.9	d	7.4	d	6.2	d
2022	M2F4	11.4	c	6.9	bc	6.6	b
	M3F6	12.6	b	7.3	b	6.8	b
	M4F8	14.3	a	7.9	a	7.2	a
	SF	10.0	d	6.6	c	6.4	b
2023	M2F4	11.7	c	7.7	ab	6.9	a
	M3F6	15.7	b	8.3	a	7.0	a
	M4F8	25.5	a	9.3	a	7.2	a
	SF	14.7	b	6.3	b	6.3	b
Significant							
Year		0.000 **		0.000 **		0.000 **	
Treatment		0.000 **		0.000 **		0.000 **	
Year × Treatment		0.000 **		0.165		0.001 **	

Note: M2F4: two rows of maize and four rows of flax, with a strip-width ratio of 80 cm:80 cm. M3F6: three rows of maize and six rows of flax, with a strip-width ratio of 120 cm:120 cm. M4F8: four rows of maize and eight rows of flax, with a strip-width ratio of 160 cm:160 cm. SM: monocropped maize. SF: monocropped flax. Different small letters indicate significant differences under the same treatment at *p* < 0.05 as determined by the LSD test; **, significant at *p* < 0.001.

**Table 4:** Phenological stage of flax at Dingxi Experimental Station, China, for 2021, 2022 and 2023.

Year	Crop	Sowing	Seeding/V3	Budding/V6	Anthesis/V12	Kernel/VT	Maturity/R1	R3	R6
2021	Flax	4/10 ^a^	5/14	6/15	6/30	7/16	8/8	-	-
	Maize	4/25	5/12	6/13	6/29	7/15	8/7	8/29	9/29
2022	Flax	4/11	5/13	6/17	7/1	7/15	8/9	-	-
	Maize	4/24	5/12	6/14	6/30	7/14	8/10	8/30	10/2
2023	Flax	4/10	5/17	6/15	7/2	7/19	8/10	-	-
	Maize	4/27	5/15	6/14	7/1	7/18	8/11	9/2	10/10

Note: ^a^, Dates are given in month/day format; phenological stage dates were recorded when 75% of the plants in a plot reached the respective growth stage. Maize phenological stages were assessed using the BBCH scale, with the designations V3, V6, V12, VT, R1, R3, and R6 corresponding to the third-leaf, sixth-leaf, twelfth-leaf, tasseling, silking, milk, and maturity stages, respectively.

## Data Availability

The raw data supporting the conclusions of this article will be made available by the authors on request.

## References

[1] Pelin T., Huseyin C., Irfan T., Asli I., Marvin S., Mojtaba K., Engin Y. (2024). The advantages of intercropping to improve productivity in food and forage production a review. Plant Prod. Sci..

[2] Glaze-Corcoran S., Hashemi M., Sadeghpour A., Jahanzad E., Afshar R.K., Liu X.B., Herbert S.J. (2020). Understanding intercropping to improve agricultural resiliency and environmental sustainability. Adv. Agron..

[3] Ryan M.R. (2021). Crops better when grown together. Nat. Sustain..

[4] Yu R.P., Dresbøll D.B., Finckh M.R., Justes E., van der Werf W., Fletcher A., Carlsson G., Li L. (2025). Intercropping: Ecosystem functioning and sustainable agriculture. Plant Soil.

[5] Maitra S., Hossain A., Brestic M., Skalicky M., Ondrisik P., Gitari H., Brahmachari K., Shankar T., Bhadra P., Palai J.B. (2021). Intercropping–A low input agricultural strategy for food and environmental security. Agronomy.

[6] Callaway R.M., Walker L.R. (1997). Competition and facilitation: A synthetic approach to interactions in plant communities. Ecology.

[7] Bertnes M.D., Callaway R. (1994). Positive interactions in communities. Trends Ecol. Evol..

[8] Li X.F., Wang Z.G., Bao X.G., Sun J.H., Yang S.C., Wang P., Wang C.B., Wu J.P., Liu X.R., Tian X. (2021). Long-term increased grain yield and soil fertility from intercropping. Nat. Sustain..

[9] Li C.J., Hoffland E., Kuyper T.W., Yu Y., Zhang C.C., Li H.G., Zhang F.S., van der Werf W. (2020). Syndromes of production in intercropping impact yield gains. Nat. Plants.

[10] Mahmoud R., Casadebaig P., Hilgert N., Alletto L., Freschet G.T., de Mazancourt C., Gaudio N. (2022). Species choice and N fertilization influence yield gains through complementarity and selection effects in cereal-legume intercrops. Agron. Sustain. Dev..

[11] van Oort P.A.J., Gou F., Stomph T.J., van der Werf W. (2020). Effects of strip width on yields in relay-strip intercropping: A simulation study. Eur. J. Agron..

[12] Wu Y.S., He D., Wang E.L., Liu X., Huth N.I., Zhao Z.G., Gong W.Z., Yang F., Wang X.C., Yong T.W. (2021). Modelling soybean and maize growth and grain yield in strip intercropping systems with different row configurations. Field Crops Res..

[13] Ajal J., Kiaer L.P., Pakeman R.J., Scherber C., Weih M. (2022). Intercropping drives plant phenotypic plasticity and changes in functional trait space. Basic Appl. Ecol..

[14] Brooker R.W., Bennett A.E., Cong W.F., Daniell T.J., George T.S., Hallett P.D., Hawes C., Iannetta P.P.M., Jones H.G., Karley A.J. (2015). Improving intercropping: A synthesis of research in agronomy, plant physiology and ecology. New Phytol..

[15] Qiang B., Yan Z., Zhang X., Ren X., Chen X. (2026). Asymmetric above- and below-ground interactions: Light availability drives root morphogenesis and productivity in maize–soybean intercropping. Soil Tillage Res..

[16] Ruillé M., Beillouin D., Prudhomme R. (2026). Ecological drivers of intercropping performance for enhanced global crop production. npj Sustain. Agric..

[17] Xu H.B., Hu J.M., Luo G.W., Wang Y.Z., Zhang Y., Rong X.M., Peng J.W., Liao C.L. (2025). Intercropping-driven maize yield gain depending on regional environments, planting conditions, and production managements: A global-scale evidence. Eur. J. Agron..

[18] Li C., Kambombe O., Chimimba E.G., Fawcett D., Brown L.A., Yu L., Gadedjisso-Tossou A., Dash J. (2023). Limited environmental and yield benefits of intercropping practices in smallholder fields: Evidence from multi-source data. Field Crops Res..

[19] Begam A., Pramanick M., Dutta S., Paramanik B., Dutta G., Patra P.S., Kundu A., Biswas A. (2024). Inter-cropping patterns and nutrient management effects on maize growth, yield and quality. Field Crops Res..

[20] Wang R.N., Sun Z.X., Zhang L.Z., Yang N., Feng L.S., Bai W., Zhang D.S., Wang Q., Evers J.B., Liu Y. (2020). Border-row proportion determines strength of interspecific interactions and crop yields in maize/peanut strip intercropping. Field Crops Res..

[21] Raza M.A., Cui L., Qin R.J., Yang F., Yang W.Y. (2020). Strip-width determines competitive strengths and grain yields of intercrop species in relay intercropping system. Sci. Rep..

[22] Zhang Y., Henke M., Li Y.M., Sun Z.P., Li W.J., Liu X.A., Li T.L. (2024). Estimating the Light Interception and Photosynthesis of Greenhouse-Cultivated Tomato Crops under Different Canopy Configurations. Agronomy.

[23] Wang J.B., Xie J.H., Li L.L., Adingo S. (2023). Review on the fully mulched ridge-furrow system for sustainable maize production on the semi-arid Loess Plateau. J. Integr. Agric..

[24] Han J., Wang Y.F., Gao Y.H., Yan B., Wang Y.Z., Lu P.N., Wang H.D., Ma X.K., Li Y.L., Wan M.L. (2026). Optimizing strip configuration improves intercropping productivity by integrating photosynthetic performance and water resource use and crop physiological adaptation. Ind. Crops Prod..

[25] Liu H.T., Chen S.H., Li B.G., Guo S., Tian J., Yao L., Lin C.W. (2022). The effect of strip orientation and width on radiation interception in maize-soybean strip intercropping systems. Food Energy Secur..

[26] Sun X.L., Zhang Y.Y., Zhang S.H., Yang N., Xia G.M., Feng L.S. (2025). Effect of strip width in maize/peanut intercropping on water use efficiency. Front. Sustain. Food Syst..

[27] Baghdadi A., Halim R.A., Othman R., Yusof M.M., Atashgahi A.R.M. (2016). Productivity, relative yield and plant growth of forage corn intercropped with soybean under different crop combination ratio. Legume Res..

[28] Chai Q., Qin A.Z., Gan Y.T., Yu A.Z. (2014). Higher yield and lower carbon emission by intercropping maize with rape, pea, and wheat in arid irrigation areas. Agron. Sustain. Dev..

[29] Wang Y.F., Qin Y.Z., Chai Q., Feng F.X., Zhao C., Yu A.Z. (2018). Interspecies interactions in relation to root distribution across the rooting profile in wheat-maize intercropping under different plant densities. Front. Plant Sci..

[30] Duchene O., Vian J.F., Celette F. (2017). Intercropping with legume for agroecological cropping systems: Complementarity and facilitation processes and the importance of soil microorganisms: A review. Agr. Ecosyst. Environ..

[31] Te X., Din A.M.U., Cui K.S., Raza M.A., Ali M.F., Xiao J.H., Yang W.Y. (2023). Inter-specific root interactions and water use efficiency of maize/soybean relay strip intercropping. Field Crops Res..

[32] Martin-Guay M.O., Paquette A., Dupras J., Rivest D. (2018). The new Green Revolution: Sustainable intensification of agriculture by intercropping. Sci. Total Environ..

[33] Mead R., Willey R.W. (1980). The Concept of a “Land Equivalent Ratio” and Advantages in Yields from Intercropping. Exp. Agric..

[34] Jin F., Wang Z.H., Zhang H.Z., Huang S.R., Chen M., Kwame T.J., Yong T.W., Wang X.C., Yang F., Liu J. (2024). Strip intercropped maize with more light interception during post-silking promotes photosynthesized carbon sequestration in the soil. Front. Plant Sci..

[35] Li S.W., van Der Werf W., Zhu J.Q., Guo Y., Li B.G., Ma Y.T., Evers J.B. (2021). Estimating the contribution of plant traits to light partitioning in simultaneous maize/soybean intercropping. J. Exp. Bot..

[36] Dai J., Shi Y.F., Zed R., Sun B.R., Shen J.B., Zhang F.S., Jin K.M. (2025). Adaptation of deep-rooted maize genotype to compacted soil: Synergy of root mechanical properties, anatomical traits, and PIEZO1 gene regulation. Soil Till. Res..

[37] Nie J.J., Li Y.N., Li Z.H., Xu S.Z., Cui Z.P., Zhan L.J., Zhang D.M., Sun L., Dai J.L., Zhang Y.J. (2026). Early cotton topping increases peanut yield without sacrificing cotton yield through reducing interspecific competition under intercropping. Field Crops Res..

[38] Dai X.P., Du X.H., Zhao S.Y., Liu H.R., Yu X.Q. (2025). Research on the advantages and optimal irrigation regime for maize-Cyperus esculentus intercropping system in northwestern Liaoning, China. Agric. Water Manag..

[39] Raza M.A., Feng L.Y., van der Werf W., Cai G.R., Bin Khalid M.H., Iqbal N., Hassan M.J., Meraj T.A., Naeem M., Khan I. (2019). Narrow-wide-row planting pattern increases the radiation use efficiency and seed yield of intercrop species in relay-intercropping system. Food Energy Secur..

[40] Yang S.Q., Zhao Y.X., Xu Y., Cui J.X., Li T., Hu Y.M., Qian X., Li Z.X., Sui P., Chen Y. (2024). Yield performance response to field configuration of maize and soybean intercropping in China: A meta-analysis. Field Crops Res..

[41] Chen X.F., Sun N., Gu Y., Zheng H.Y., Li J.F., Wu C.S., Wang Z.M. (2020). Photosynthetic and Chlorophyll Fluorescence Responses in Maize and Soybean Strip Intercropping System. Int. J. Agric. Biol..

[42] Moreira A., Moraes L.A.C., Schroth G., Mandarino J.M.G. (2015). Effect of nitrogen, row spacing, and plant density on yield, yield components, and plant physiology in soybean-wheat intercropping. Agron. J..

[43] Kou H.T., Liao Z.Q., Liu Y.Y., Zhang H., Lai Z.L., Yu J., Li Z.J., Fan J.L. (2025). Light distribution, interception and use efficiency of drip-fertigated maize-soybean strip intercropping systems under various row configuration conditions. Plant Cell Environ..

[44] Luo K., Yuan X.T., Zhang K.J., Fu Z.D., Lin P., Li Y.L., Li Y.Z., Pu T., Wang X.C., Yang W.Y. (2025). Soybean variety improves canopy architecture and light distribution to promote yield formation in maize–soybean strip intercropping. Plant Cell Environ..

[45] Echarte L., Della Maggiora A., Cerrudo D., Gonzalez V.H., Abbate P., Cerrudo A., Sadras V.O., Calviño P. (2011). Yield response to plant density of maize and sunflower intercropped with soybean. Field Crops Res..

[46] Mahallati M.N., Koocheki A., Mondani F., Feizi H., Amirmoradi S. (2015). Determination of optimal strip width in strip intercropping of maize (*Zea mays* L.) and bean (*Phaseolus vulgaris* L) in Northeast Iran. J. Clean. Prod..

[47] Doubi B.T.S., Kouassi K.I., Kouakou K.L., Koffi K.K., Baudoin J.P., Zoroa B.I.A. (2016). Existing competitive indices in the intercropping system of Manihot esculenta Crantz and Lagenaria siceraria (Molina) Standley. J. Plant Interact..

[48] Luo C.L., Duan H.X., Wang Y.L., Liu H.J., Xu S.X. (2024). Complementarity and competitive trade-offs enhance forage productivity, nutritive balance, land and water use, and economics in legume-grass intercropping. Field Crops Res..

[49] Dzvene A.R., Tesfuhuney W.A., Walker S., Ceronio G. (2023). Planting time and stand density effect on radiation interception and use efficiency of maize and sunn hemp intercropping in semi-arid South Africa. Agric. For. Meteorol..

[50] Li L., Sun J., Zhang F., Li X., Yang S., Rengel Z. (2001). Wheat/maize or wheat/soybean strip intercropping yield advantage and interspecific interactions on nutrients. Field Crops Res..

[51] Dhima K.V., Lithourgidis A.S., Vasilakoglou I.B., Dordas C.A. (2007). Competition indices of common vetch and cereal intercrops in two seeding ratio. Field Crops Res..

